# Understanding the mechanism of twenty-five ingredient decoction for setting a fracture in the treatment of fractures based on network pharmacology

**DOI:** 10.1097/MD.0000000000032864

**Published:** 2023-02-03

**Authors:** Zenghui Tian, Yanke Hao, Mingliang Wang, Yingying Li, Kaiying Cui, Pengfei Hou, Xiaoying Wang, Dengwan Lv, Jie Shi

**Affiliations:** a College of First Clinical Medical, Shandong University of Traditional Chinese Medicine, Jinan, P.R. China; b Affiliated Hospital of Shandong University of Traditional Chinese Medicine, Jinan, P.R. China; c Rizhao Hospital of Traditional Chinese Medicine, Rizhao, P.R. China; d Jinan Vocational College of Nursing, Jinan, P.R. China; e College of Traditional Chinese Medicine, Shandong University of Traditional Chinese Medicine, Jinan, P.R. China.

**Keywords:** fracture, mechanism of action, network pharmacology, twenty-five ingredient decoction for setting a fracture

## Abstract

To study the mechanism of 25 ingredient decoction for setting a fracture (TDSF) in fracture treatment using network pharmacology. The TCMSP, BATMAN-TCM, HERB, and Uniprot protein databases were used to identify the active ingredients and targets of TDSF. Fracture-related targets were collected from the gene cards and the online mendelian inheritance in man databases. The acquisition of common genes of active compounds of TDSF and disease fractures was carried out using the Venny software. The Cytoscape 3.7.1 software and String database were used to construct a network diagram of drug-active ingredient-target-disease and the main core targets were obtained by protein interaction analysis. The Metascape platform was used to perform gene oncology functional and Kyoto encyclopedia of genes and genomes pathway enrichment analyses for common drug-disease targets. A total of 311 active ingredients and 348 targets were associated with TDSF, with 5197 targets related to fractures and 224 common targets between the 2 keywords. Key targets included serine/threonine protein kinase 1, tumor necrosis factor, interleukin 6, tumor protein 53, and vascular endothelial growth factor. Important roles of the following pathway were identified: cancer, lipid, and atherosclerosis; AGE-RAGE signaling pathway in diabetic complications; chemical carcinogenesis – receptor activation; PI3K -Akt signaling pathway; platinum drug resistance; cAMP signaling pathway; transcriptional mis regulation in cancer; serotonergic synapse; and malaria. TDSF mainly treats fractures by acting on multiple targets, such as serine/threonine protein kinase 1, tumor necrosis factor, interleukin 6, tumor protein 53, and vascular endothelial growth factor, and regulating the PI3K/AKT and cAMP signaling pathways.

## 1. Introduction

The incidence of fractures between 1989 and 2013 was 2% to 3%, and fractures are the most common trauma and the main cause of disability.^[1]^ Despite economic growth and advancement in science and technology, the incidence of nonunion is still 5% to 10% in patients with fractures who can receive active treatment.^[2]^ Patients with nonunion mostly require surgery. Although the surgical procedure may result in the healing of the fracture, the patient limb function may be impacted and the patient economical and psychological conditions can be challenged.^[3]^ Therefore, selecting a method that can promote fracture healing, shorten the fracture healing duration, and reduce the incidence of nonunion is an attractive and popular topic in trauma orthopedics.

According to traditional Chinese medicine, fracture healing is a process consisting of “removal of blood stasis, regeneration, and osseointegration” and the treatment of fractures is divided into 3 stages. In the initial stage, fracture causes displacement and soft tissue damage, characterizes by local bleeding to form hematoma, congested tissues, and edema, and results in swelling. In the Qi stagnation and blood stasis stage, treatment focuses on promoting blood circulation and Qi, removing blood stasis, and relieving pain. In the middle stage, the local hematoma is absorbed, edema is relieved, soft tissues are repaired, and the callus starts to form again. These changes decrease local swelling and pain. In the late stage, treatment mainly focuses to strengthen the bones and tendons and nourishing the liver and kidneys.^[4,5]^ Twenty-five ingredient decoction for setting a fracture (TDSF) is a classic prescription for fracture treatment recorded by Wei Yilin, a physician in the Yuan Dynasty, in “Shiyi Dexiao Prescription,” which is the representative prescription of “Shaolin” traumatology school to treat fractures. TDSF is made of Angelicae Dahuricae Radix, Lycii Cortex, Diverse Wormwood Herbs, Angelicae Sinensis Radix, Paeoniae Radix Rubra, Paeoniae Radix Alba, Pharbitidis Semen, Rehmanniae Radix, Chuanxiong Rhizoma, Achyranthis Bidentatae Radix, Olibanum, Myrrha, Psoraleae Fructus, Akebiae Caulis, Aucklandiae Radix, Pygosternonis Herba, Pyritum, Equiseti Hiemalis Herba, Cinnamomi Cortex, Notopterygii Rhizoma Et Radix, Angelicae Pubescentis Radix, Pinelliae Rhizoma, Drynariae Rhizoma, Aconiti Kusnezoffii Radix, and Aconiti Radix. This prescription uses blood-activating and qi-regulating medicines in combination, mainly for activating blood, healing wounds, activating qi, and relieving pain. Further, the prescription expels wind and dampness, nourishes the liver and kidneys, and strengthens muscles and bones.^[6,7]^

Although TDSF has achieved good results in clinical applications,^[6]^ the mechanism of action of TDSF in the treatment of fractures is still unclear. This study used network pharmacology to screen out the main active ingredients and potential targets in prescriptions, predict and explore the mechanism of action of TDSF in the treatment of fractures, and provide theoretical support for future development and utilization.

## 2. Materials and methods

### 2.1. Active ingredients and potential targets of TDSF

After setting the core screening parameters of pharmacokinetics (absorption, distribution, metabolism, and excretion), with oral bioavailability of ≥ 30% and drug-likeness of ≥ 0.18, active ingredients of TDSF were collected using the TCMSP (http://tcmspw.com/tcmsp.php),^[8]^ BATMAN-TCM (http://bionet.ncpsb.org/batman-tcm/), and HERB (http://herb.ac.cn/) databases. The obtained protein targets were entered into the Uniprot protein database (https://www.uniprot.org/) and BATMAN-TCM database for gene name specifications, with species set as “Homo sapiens.”

### 2.2. Screening of fracture-related targets

Using “fracture” as the keyword, the Gene Cards (http://www.genecards.org) and the online mendelian inheritance in man (OMIM) (http://www.omim.org) databases were searched to collect information about fracture targets, summarize all targets, and remove duplicate items to obtain fracture-related targets.

### 2.3. Active ingredient-target-disease network graph construction and analysis

The targets of the active ingredients of TDSF were mapped with fracture-related targets to form a Venn diagram, and the common drug-disease target (potential targets of TDSF for the treatment of fractures) was obtained. The Cytoscape 3.7.1 software was used to correlate active ingredients, intersect targets and diseases, and build a network diagram of active ingredients-targets-diseases.

### 2.4. Construction and analysis of protein-protein interaction (PPI) network

The drug-disease common targets were uploaded to the String database (https://string-db.org/), with species and minimum interaction threshold set as “Homo sapiens” and “medium confidence > 0.4,” respectively, to construct a protein-protein interaction (PPI) network graph. The Cytoscape 3.7.1 software was used for topology analysis and the “CytoNCA” plug-in was used to analyze the degree value, betweenness centrality (BC), closeness centrality (CC), and local average connectivity (LAC). Further, the main core targets were screened according to their medians.

### 2.5. Gene oncology (GO) functional and Kyoto encyclopedia of genes and genomes (KEGG) pathway enrichment analysis

The drug-disease intersection targets were imported into the Metascape platform (http://metascape.org), and the species “homo sapiens” was selected for gene oncology (GO) and Kyoto encyclopedia of genes and genomes (KEGG) pathway enrichment analyses, and the obtained results were visualized.

## 3. Results

### 3.1. Active ingredients and potential targets of drugs

A total of 311 active ingredients were obtained in TDSF, including Angelicae Dahuricae Radix (22), Lycii Cortex (13), Diverse Wormwood Herb (5), Angelicae Sinensis Radix (2), Paeoniae Radix Rubra (29), Paeoniae Radix Alba (13), Pharbitidis Semen (22), Rehmanniae Radix (3), Chuanxiong Rhizoma (7), Achyranthis Bidentatae Radix (20), Olibanum (8), Myrrha (44), Psoraleae Fructus (12), Akebiae Caulis (8), Aucklandiae Radix (6), Pygosternonis Herba (11), Pyritum (1), Equiseti Hiemalis Herba (9), Cinnamomi Cortex (10), Notopterygii Rhizoma Et Radix (15), Angelicae Pubescentis Radix (9), Pinelliae Rhizoma (13), Drynariae Rhizoma (18), Aconiti Kusnezoffii Radix (8), and Aconiti Radix (3). Among them, 29 active ingredients shared by the drugs (Table 1). These 311 active ingredients were matched with the Uniprot protein and BATMAN-TCM databases to obtain drug-gene targets. After removing duplicate items, 348 effective active targets were identified.

**Table 1:** Active ingredients shared by the drug.

Active ingredients	Drugs
Stigmasterol	Psoraleae Fructus, Angelicae Dahuricae Radix, Lycii Cortex, Angelicae Sinensis Radix, Paeoniae Radix Alba, Achyranthis Bidentatae Radix, Olibanum, Akebiae Caulis, Aucklandiae Radix, Pinelliae Rhizoma, Drynariae Rhizoma
Mandenol	Angelicae Dahuricae Radix, Chuanxiong Rhizoma, Equiseti Hiemalis Herba
Ammidin	Angelicae Dahuricae Radix, Notopterygii Rhizoma Et Radix, Angelicae Pubescentis Radix
isoimperatorin	Angelicae Dahuricae Radix, Notopterygii Rhizoma Et Radix, Angelicae Pubescentis Radix
Cnidilin	Angelicae Dahuricae Radix, Notopterygii Rhizoma Et Radix
Ethyl oleate (NF)	Angelicae Dahuricae Radix, Paeoniae Radix Rubra
beta-sitosterol	Angelicae Dahuricae Radix, Lycii Cortex, Diverse Wormwood Herb, Angelicae Sinensis Radix, Paeoniae Radix Rubra, Paeoniae Radix Alba, Pharbitidis Semen, Achyranthis Bidentatae Radix, Myrrha, Akebiae Caulis, Equiseti Hiemalis Herba, Notopterygii Rhizoma Et Radix, Angelicae Pubescentis Radix, Pinelliae Rhizoma, Drynariae Rhizoma
CLR	Angelicae Dahuricae Radix, Lycii Cortex
Phellopterin	Angelicae Dahuricae Radix, Notopterygii Rhizoma Et Radix
hederagenin	Lycii Cortex, Akebiae Caulis
luteolin	Diverse Wormwood Herb, Equiseti Hiemalis Herba, Drynariae Rhizoma
ellagic acid	Paeoniae Radix Rubra, Myrrha
paeoniflorgenone	Paeoniae Radix Rubra, Paeoniae Radix Alba
Lactiflorin	Paeoniae Radix Rubra, Paeoniae Radix Alba
paeoniflorin_qt	Paeoniae Radix Rubra, Paeoniae Radix Alba
baicalein	Paeoniae Radix Rubra, Achyranthis Bidentatae Radix, Pinelliae Rhizoma
Baicalin	Paeoniae Radix Rubra, Achyranthis Bidentatae Radix, Pinelliae Rhizoma
sitosterol	Paeoniae Radix Rubra, Paeoniae Radix Alba, Pharbitidis Semen, Chuanxiong Rhizoma, Aucklandiae Radix, Notopterygii Rhizoma Et Radix
Spinasterol	Paeoniae Radix Rubra, Paeoniae Radix Rubra
(+)-catechin	Paeoniae Radix Rubra, Paeoniae Radix Alba, Drynariae Rhizoma
benzoyl paeoniflorin	Paeoniae Radix Rubra, Paeoniae Radix Alba
Albiflorin_qt	Paeoniae Radix Rubra, Paeoniae Radix Alba
Mairin	Paeoniae Radix Alba, Aucklandiae Radix
kaempferol	Paeoniae Radix Alba, Paeoniae Radix Rubra, Equiseti Hiemalis Herba, Drynariae Rhizoma
poriferasta-7,22E-dien-3beta-ol	Paeoniae Radix Rubra, Myrrha
quercetin	Paeoniae Radix Rubra, Myrrha, Pygosternonis Herba, Equiseti Hiemalis Herba
Diop	Pygosternonis Herba, Equiseti Hiemalis Herba
nodakenin	Notopterygii Rhizoma Et Radix, Angelicae Pubescentis Radix
hypaconitine	Aconiti Kusnezoffii Radix, Aconiti Radix

### 3.2. Screening of fracture-related targets

Using “fracture” as the keyword, 5181 and 22 disease genes were retrieved from the Gene Cards and OMIM databases, respectively. The retrieval results of the Gene Cards and OMIM databases were summarized and a total of 5197 fracture targets were obtained after deleting duplicate items.

### 3.3. Active ingredient-target-disease network graph construction and analysis

**Figure 1.**
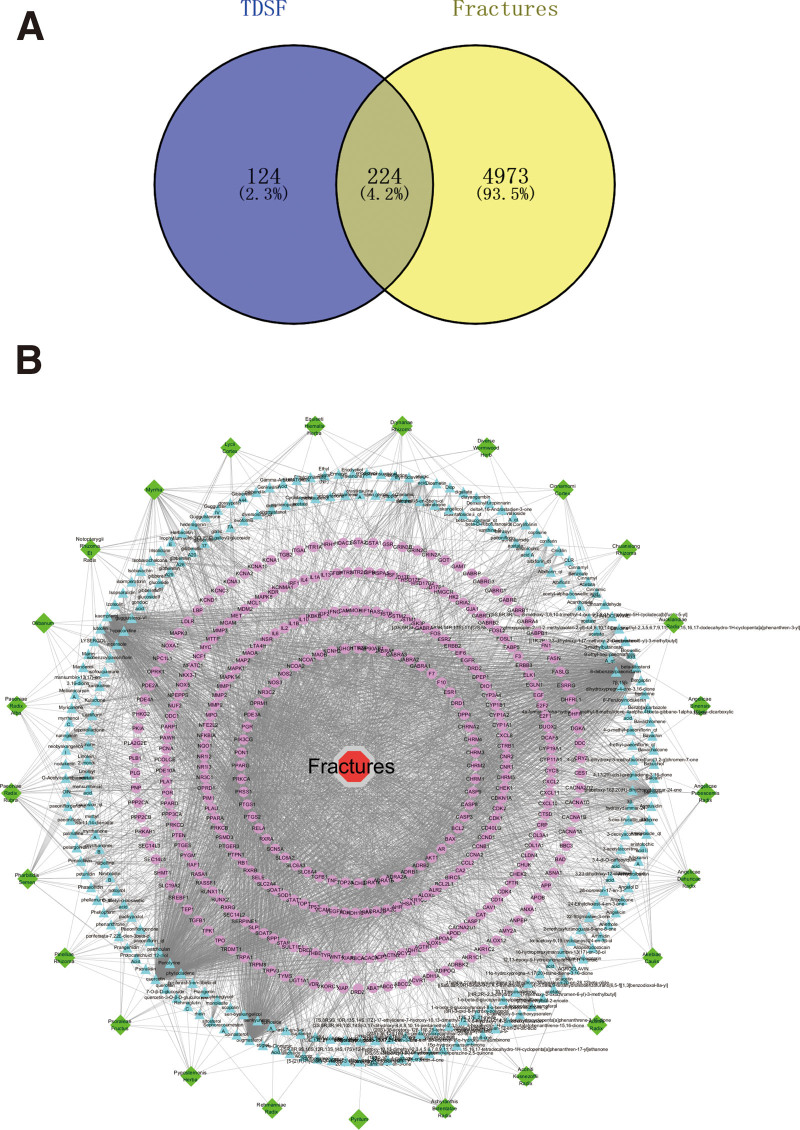
Active ingredient-target-disease network graph construction and analysis. (A) Screening of common targets of TDSF and disease. Dark blue circles indicate targets of active compounds present in TDSF, whereas yellow circles represent targets of disease in fractures. (B) Regulatory networks between TDSF and fractures. Green, blue, purple, and red graphs represent the composition of TDSF, active ingredients, potential targets, and diseases, respectively. TDSF = twenty-five ingredient decoction for setting a fracture.

The effective active target proteins of the drug and fracture were entered into the VENNY 2.1 system (https://bioinfogp.cnb.csic.es) and a Venn diagram was prepared (Fig. 1A). A total of 224 intersecting gene targets were identified and considered potential targets of TDSF for fracture treatment. The active ingredients and effective active targets of the drug and 224 potential targets were imported into the Cytoscape 3.7.1 software to construct an active ingredient-target-disease network diagram (Fig. 1B). The network contained 602 targets and 3908 edges. Further, TDSF seemed to show multi-target action in the treatment of fractures.

### 3.4. PPI network construction and core target screening

**Figure 2.**
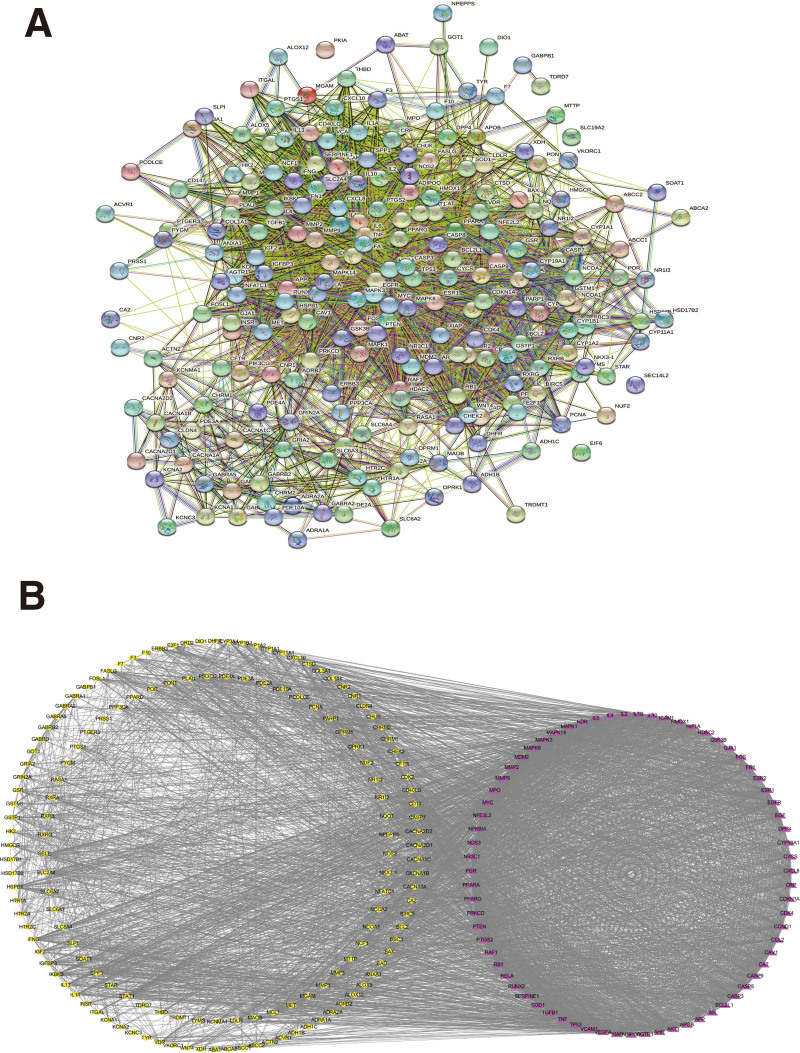
PPI network construction and core target screening. (A) The PPI network was constructed from the STRING platform. (B) The network graph was obtained by screening according to the following conditions: degree ≥ 29, LAC ≥ 17.942, BC ≥ 0.002, and CC ≥ 0,494. Red and yellow nodes represent conditional and unconditional target proteins, respectively. BC = betweenness centrality, CC = closeness centrality, LAC = local average connectivity, PPI = protein-protein interaction.

**Figure 3.**
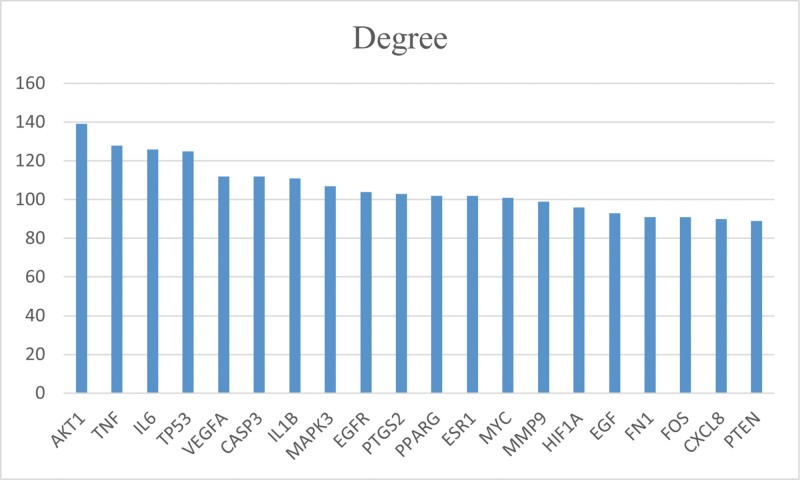
Bar graphs showing the top 20 target proteins ranked according to the value of the degree.

The 224 TDSF targets were imported into the STRING platform to construct a PPI network (Fig. 2A). The network consisted of 224 nodes and 4135 edges. The results were imported into Cytoscape 3.7.1 software, and the values of degree, BC, CC, and LAC were calculated using the CytoNCA plug-in. The median of each value (degree ≥ 29, LAC ≥ 17.942, BC ≥ 0.002, and CC ≥ 0,494) was used as the standard to filter, and the network graph was obtained (Fig. 2B). Here, we considered that the higher the number of edges connected to a node, the more important the biological function of that node in the network and was considered as a hub gene. A bar graph was created for the target proteins that met the conditions, and the top 20 target proteins were ranked according to the degree value (Fig. 3). The top 5 targets, vascular endothelial growth factor (VEGFA), tumor protein 53 (TP53), interleukin 6 (IL6), tumor necrosis factor (TNF), and serine/threonine protein kinase 1 (AKT1), were ranked in descending order of degree value. These top 5 targets may be the key targets for the treatment of fractures.

### 3.5. GO functional and KEGG pathway enrichment analyses

**Figure 4.**
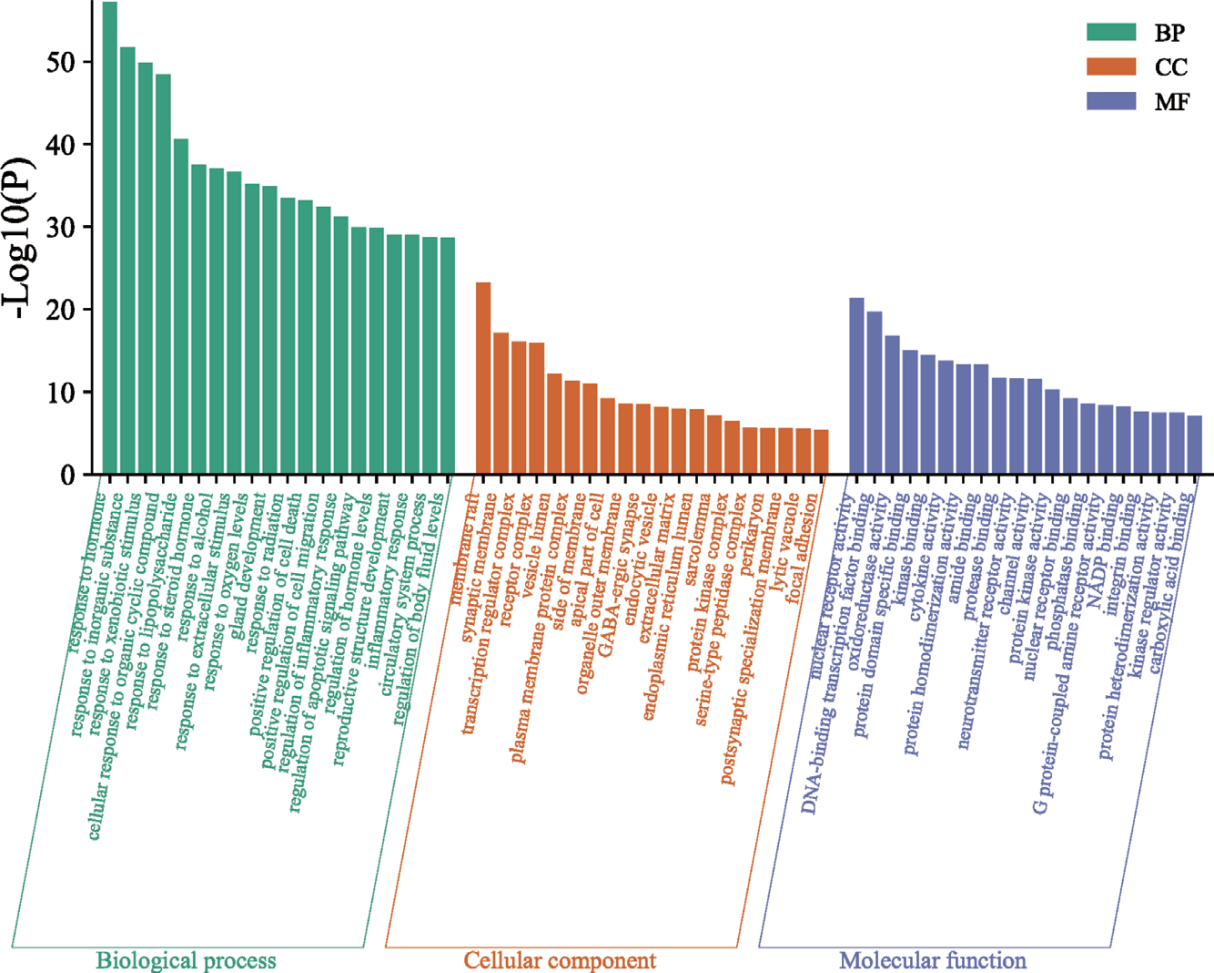
Gene Ontology functional enrichment analyses of common genes. Significant enrichment of common genes was observed in molecular function, cellular component, and biological process categories (the top 20 GO terms for each category are listed). The X-axis represents 3 GO terms: molecular function, cellular component, and biological process. The y-axis represents -log10(p) of GO terms. The -log (P) value of GO terms is shown numerically above the bar chart. GO = gene oncology.

**Figure 5.**
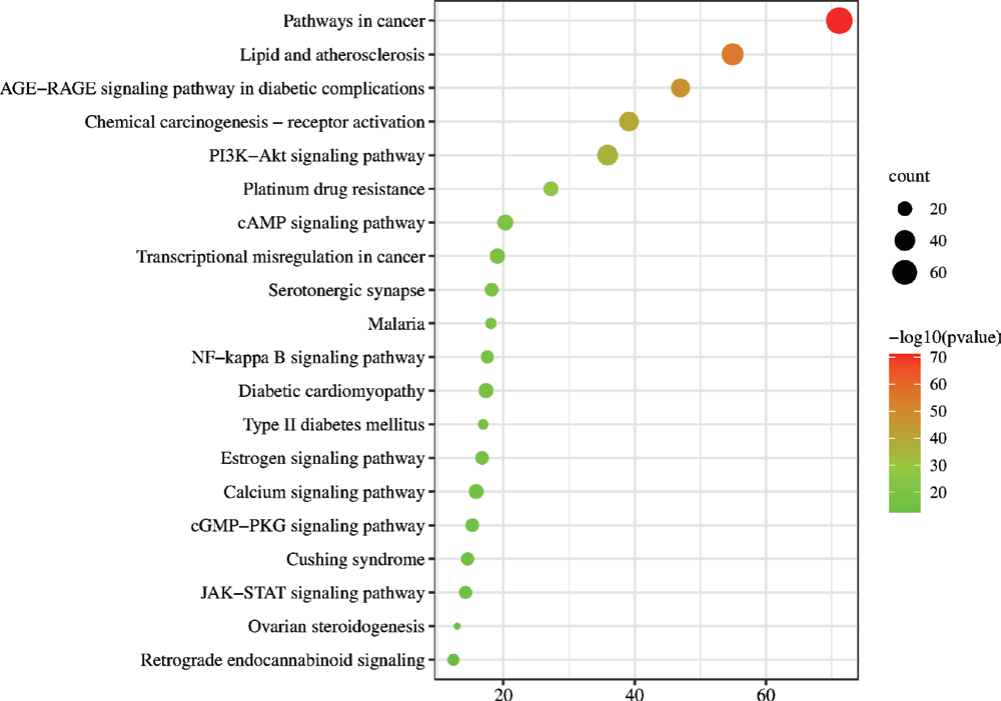
KEGG pathway enrichment analysis and a bubble graph. The number of genes enriched in each pathway is represented by the size of the circle from green to red representing the change in the *P* value. KEGG = Kyoto encyclopedia of genes and genomes.

The potential targets of TDSF for fracture treatment were uploaded to the Metascape platform for GO function (Fig. 4) and KEGG pathway enrichment analyses, and a bubble chart was prepared (Fig. 5).

The GO enrichment analysis showed that the treatment of fractures with TDSF mainly regulates the response to hormones, inorganic substances, xenogeneic biological stimuli, organic cyclic compounds, and lipopolysaccharides. In terms of molecular function, TDSF mainly regulates the activity of nuclear receptors, the binding of DNA transcription factors, the activity of oxidoreductases, the specific binding of protein domains, and the binding of kinases. At the cellular component level, TDSF intervenes in structures such as membrane rafts, synaptic membranes, transcription factor complexes, receptors, and the vesicle lumen.

The KEGG pathway enrichment analysis of potential targets and the analysis of bubble plots identified the following pathways/genes/conditions closely related to fracture: cancer, lipid and atherosclerosis, AGE-RAGE signaling pathway in diabetic complications, chemical carcinogenesis – receptor activation, PI3K-Akt signaling pathway, platinum drug resistance, cAMP signaling pathway, transcriptional misregulation in cancer, serotonergic synapse, and malaria.

## 4. Discussion

Network pharmacology was used to analyze the mechanism of action of TDSF in the treatment of fractures, and the gene targets of active ingredients of TDSF were obtained from the TCMSP, BATMAN-TCM, HERB, and Uniprot protein databases. The Gene Cards and OMIM databases were used to obtain the gene targets of fractures and the intersection of the 2 databases yielded a total of 224 common gene targets. Potential targets for the treatment of diseases were screened using the PPI analysis of common targets. In addition, GO and KEGG pathway enrichment analyses demonstrated that TDSF mainly functions through the PI3K/AKT and cAMP signaling pathways, and the potential targets AKT1, TNF, IL6, TP53, and VEGFA are enriched in these 2 pathways. Furthermore, it was found that TDSF mainly acts on AKT1, TNF, IL6, TP53, VEGFA, and other targets and regulates the PI3K/AKT and cAMP signaling pathways to treat fractures.

Recent studies have confirmed that the PI3K/AKT and cAMP signaling pathways play important regulatory roles in fracture healing. After PI3K is activated, it can bind to the PH domain-containing signaling protein AKT and phosphoinositide-dependent protein kinase (PDK1) in cells through the secondary messenger PIPa and promoting the activation of AKT, thereby regulating cell proliferation, movement, growth, survival, and metabolic function.^[9,10]^ Furthermore, cell proliferation is regulated by activating the PI3K/AKT signaling pathway to promote fracture healing. Bone marrow-derived mesenchymal stem cells (BM-MSCs) are important regulators of fracture healing, and angiogenesis is a key process in bone healing. Yang et al used miR-29b-3pKD-BMSCs in vitro with a human umbilical cord in a co-culture of venous endothelial cells. Activation of the PI3K/AKT pathway was found to promote human umbilical cord in a co-culture of venous endothelial cells proliferation, migration, angiogenesis, and ultimately fracture healing.^[11]^ Xu et al^[12]^ studied the regulatory effect of low-dose IL-34 on osteoblastogenesis and osteoclastogenesis and found that low-dose IL-34 regulates the osteogenesis of hBMSCs by activating the PI3K/AKT pathway and enhances fracture healing. Recent studies have shown that the PI3K/AKT signaling pathway not only regulates cell proliferation and growth but also enhances communication between bone cells and promotes fracture healing.^[13]^

The cAMP signaling pathway channels extracellular signals to G protein-coupled receptors, resulting in changes in the level of intracellular secondary messenger cAMP and eliciting cellular responses. cAMP plays a role in the control of various cellular processes. Xi et al^[14]^ studied the relationship between the flavonoid extract of Epimedium Herba (TFE) and peak bone mass in young rats and found that the extract promotes bone formation by activating the cAMP pathway in vivo. Xie et al^[15]^ also confirmed that the activation of the cAMP signaling pathway affects the differentiation of osteoblasts. Dong Wantao et al^[16]^ conducted in vivo experiments with Xiaoding ointment, observed pathological changes in callus tissue under a light microscope, and detected the expression of the cAMP protein in callus tissue by immunohistochemistry. They found that the Xiaoding ointment promoted fracture healing by regulating the cAMP signaling pathway.

## 5. Conclusions

In conclusion, through a preliminary analysis of network pharmacology, we identified the effective active ingredients, key targets, and pathways of TDSF during fracture treatment. The treatment of fractures using TDSF is a complex process involving multiple components, targets, and pathways. TDSF may be involved in the therapeutic process of fractures via the PI3K/AKT and cAMP signaling pathways through key targets, such as AKT1, TNF, IL6, TP53, and VEGFA.

## Author contributions

**Conceptualization:** Yanke Hao, Mingliang Wang.

**Data curation:** Zenghui Tian, Mingliang Wang.

**Formal analysis:** Yanke Hao.

**Investigation:** Xiaoying Wang, Yingying Li.

**Methodology:** Zenghui Tian, Mingliang Wang.

**Project administration:** Yanke Hao, Mingliang Wang.

**Resources:** Yingying Li, Pengfei Hou.

**Software:** Zenghui Tian, Mingliang Wang, Dengwan LV.

**Supervision:** Kaiying Cui, Pengfei Hou.

**Validation:** Dengwan Lv, Kaiying Cui, Jie Shi.

**Writing – original draft:** Zenghui Tian, Mingliang Wang.

**Writing – review & editing:** Yanke Hao, Mingliang Wang.

## References

[1] Trajanoska K Schoufour JD de Jonge EAL. Fracture incidence and secular trends between 1989 and 2013 in a population based cohort: the Rotterdam Study. Bone. 2018;114:116–24. 29885926 10.1016/j.bone.2018.06.004

[2] Wang D Liu Y Lv W. Repetitive brief ischemia accelerates tibial shaft fracture healing: a 5-years prospective preliminary clinical trial (PCT). BMC Musculoskelet Disord. 2021;22:631. 34284739 10.1186/s12891-021-04515-y PMC8293516

[3] Zhang J Wang D. “ The study of the mechanism of Xuduan promoting fracture healing based on network pharmacology and molecular docking.”. J Capital Med Univ. 2022;43:275–83.

[4] Duan C Zhou XJ Che GL. “ Clinical effect of C3 colles fracture treated by manual reduction and small splint fixation combined with traditional Chinese medicine in three-stage dialectical external treatment”. J Emerg Tradit Chin Med. 2021;30:1562–6.

[5] Yu ZY Wen JM Zhang YF. “ Application and challenges of the TCM three phases syndrome differentiation system of fracture after modern orthopaedics surgery”. China J Tradit Chin Med Pharm. 2017;32:4592–4.

[6] Jiang XP. “ External application of twenty-five ingredient decoction for set a fracture in the treatment of 52 cases of radial fracture with extension.”. J Sichuan Tradit Chin Med. 2012;30:104.

[7] Lu WH. “ Analysis on internal treatment of fractures in ‘Shiyi Dexiao Fang’ by Wei Yilin, a physician in Xujiang.”. J Clin Med. 2018;5:55 + 57.

[8] Ru J Li P Wang J. TCMSP: a database of systems pharmacology for drug discovery from herbal medicines. J Cheminf. 2014;6:13. 10.1186/1758-2946-6-13 PMC4001360 24735618

[9] Cantley LC. The phosphoinositide 3-kinase pathway. Science. 2002;296:1655–7. 12040186 10.1126/science.296.5573.1655

[10] Kumar M Bansal N. Implications of phosphoinositide 3-Kinase-Akt (PI3K-Akt) pathway in the pathogenesis of Alzheimer’s disease. Mol Neurobiol. 2022;59:354–85. 34699027 10.1007/s12035-021-02611-7

[11] Yang J Gao J Gao F. Extracellular vesicles-encapsulated microRNA-29b-3p from bone marrow-derived mesenchymal stem cells promotes fracture healing via modulation of the PTEN/PI3K/AKT axis. Exp Cell Res. 2022;412:113026. 35026284 10.1016/j.yexcr.2022.113026

[12] Xu J Fu L Bai J. Low-dose IL-34 has no effect on osteoclastogenesis but promotes osteogenesis of hBMSCs partly via activation of the PI3K/AKT and ERK signaling pathways. Stem Cell Res Ther. 2021;12:268. 33947456 10.1186/s13287-021-02263-3 PMC8097863

[13] Liu Y Duan M Guo D. PDGF-AA promotes cell-to-cell communication in osteocytes through PI3K/Akt signaling pathway. Acta Biochim Biophys Sin. 2021;53:1640–9. 34586354 10.1093/abbs/gmab136

[14] Xi HR Ma HP Yang FF. Total flavonoid extract of Epimedium herb increases the peak bone mass of young rats involving enhanced activation of the AC10/cAMP/PKA/CREB pathway. J Ethnopharmacol. 2018;223:76–87. 29783019 10.1016/j.jep.2018.05.023

[15] Xie W Li F Han Y. Neuropeptide Y1 receptor antagonist promotes osteoporosis and microdamage repair and enhances osteogenic differentiation of bone marrow stem cells via cAMP/PKA/CREB pathway. Aging (Milano). 2020;12:8120–36. 10.18632/aging.103129 PMC7244071 32381754

[16] Dong WT Song M Chen BX. “Effects of Xiaoding Ointment on COX-2/PGE2/cAMP signal pathway expression in fracture heading.”. Chin Tradit Patent Med. 2018;40:20–6.

